# Quality and reliability of Chinese short videos on TikTok related to chronic renal failure: cross-sectional study

**DOI:** 10.3389/fpubh.2025.1652579

**Published:** 2025-11-12

**Authors:** Kai Wang, Xianjiao Tan, Pei Liu

**Affiliations:** Department of Information, Daping Hospital, Army Medical University, Chongqing, China

**Keywords:** chronic renal failure, TikTok, video quality, DISCERN tool, Global Quality Score, content analysis, online health information, health information quality

## Abstract

**Background:**

Chronic renal failure is projected to be one of the fastest-growing causes of death among non-communicable diseases by 2040. TikTok has emerged as a major platform for disseminating health-related videos. However, the reliability and quality of Chinese videos related to chronic renal failure on TikTok remain unclear. We systematically searched and screened videos related to chronic renal failure from the Chinese version of TikTok. Two independent raters assessed the reliability and quality of the videos using two validated evaluation tools: the DISCERN instrument and the Global Quality Score (GQS). Moreover, the correlation between the reliability and quality of the videos and their characteristics (duration, likes, comments, shares, and number of followers) was further investigated.

**Results:**

After searching and screening, a total of 78 eligible videos were ultimately included for analysis. According to their sources, 94.87% were uploaded by medical professionals. The median DISCERN and GQS scores were 39 (IQR 37–46.25) and 3 (IQR 2.75–4), respectively, indicating that videos related to chronic renal failure on TikTok were unreliable and of mediocre quality, mainly at poor (42.31%) and moderate (44.87%) categories. The reliability and quality of the videos were positively correlated with video duration (*r* = 0.384, *p* = 0.001; *r* = 0.469, *p* < 0.01) and showed no statistically significant correlation with popularity or number of followers. Consequently, due to their unreliability and low quality, these Chinese videos related to chronic renal failure on TikTok cannot provide patients with accurate assessments and are unsuitable as a source of medical knowledge.

## Introduction

Chronic renal failure is characterized by long-term kidney damage resulting from conditions like diabetes, hypertension, and glomerulonephritis, along with other complex causes (1, 2). The global prevalence of chronic renal failure stands at 9.1%, with reported deaths reaching 1.2 million and mortality rates rising by 41.4% (3). Furthermore, studies have concluded that by 2040, the number of years of life lost due to premature deaths from CKD will be twice as high as in 2016, and the number of deaths will be three times that of 2016, making it one of the fastest-growing causes of death from non-communicable diseases (4). An estimated 82 million adults in China had chronic renal failure, with a prevalence of 8.2%. However, only 10.0% of these patients were aware that they had the disease (5), making chronic renal failure often referred to as the “silent killer.”

Treatment for chronic renal failure involves dietary adjustments and medication. The condition worsens over time due to multiple factors, and when it progresses to its terminal stage, it is known as uremia. In the uremic stage, it may even require peritoneal dialysis, hemodialysis (6), or kidney transplantation (7). The disease is more common in the older adults, rural residents, and individuals with lower education or income levels (5). As chronic renal failure is irreversible, patients must have accurate knowledge about the disease to enable early detection, diagnosis, and treatment to slow kidney damage. Patients’ lack of understanding about their condition and inadequate anticipation of its progression increase the financial burden and time commitment required for subsequent treatment. Previous studies have employed substantially greater influence of machine learning (ML) models (8) to provide more accurate and timely assessments of patients’ kidney health, enabling individuals at risk or already affected by chronic renal failure to pursue prevention and treatment (9).

The Internet and social media have significantly altered how people access health information, with more patients researching their conditions online before seeking medical help (10). However, social media also faces challenges regarding the credibility and authenticity of information. Some publishers exploit social media features to exaggerate facts to attract attention or profit, resulting in the spread of misinformation (11). TikTok is available in 160 countries and has over 1.1 billion users, making it the fastest-growing social media platform. In China, TikTok has well over 700 million users and 400 million daily active users (12). In 2022, TikTok’s monthly video views reached 400 billion, with single-day views exceeding 120 million in 2023. The platform’s growing user base and video content have resulted in varying quality due to a lack of content filtering. Incorrect health information can mislead patients, leading to poor decision-making and increased health risks.

Previous studies have evaluated the quality and reliability of TikTok videos related to various diseases, such as uterine fibroids (13), stroke prevention (14), hypertrophic scarring (15), lung cancer (16), brain tumor (17), breast cancer (18), gastric cancer (19), cervical cancer (20), heart failure (21). Existing research indicates that the information quality and reliability of disease-related videos on TikTok are generally unsatisfactory. However, content related to cosmetic surgery demonstrates satisfactory quality and reliability (22). We have discovered a large number of Chinese videos related to chronic renal failure on TikTok. TikTok has become a significant channel for the public to access knowledge about chronic renal failure, but the quality and reliability of these videos remain unstudied. Given that misinformation about chronic renal failure can lead patients to develop incorrect perceptions of the disease, thereby delaying treatment or even resulting in incorrect treatment. The adverse impact on health outcomes exacerbates medical conflicts arising from differing perceptions of disease between clinicians and patients, thereby eroding patients’ trust in healthcare (23). Therefore, assessing the quality and reliability of information about chronic renal failure on TikTok is crucial.

This study aims to evaluate the quality and reliability of videos related to chronic renal failure on TikTok and further investigate the correlation between these attributes and video characteristics (duration, likes, comments, shares, and number of followers). The contributions of this study are as follows:

1. Addressing the existing academic gap in this field.
2. Evaluating the quality and reliability of the videos related to chronic renal failure on TikTok to help the public make more appropriate choices regarding such content.
3. Providing accurate and reliable disease information to enable high-risk individuals to identify risk factors, recognize early warning signs, and undergo regular screenings — thus facilitating earlier intervention and treatment.
4. High-quality health education videos can enhance public awareness of chronic renal failure, thereby alleviating patients’ anxiety and fear. Reducing the knowledge gap about disease can establish more effective doctor-patient communication, which helps improve treatment adherence, slow the progression of kidney function decline, and potentially prevent the condition from advancing to the uremic stage, thereby reducing the economic burden.
5. Providing recommendations and guidance for video platforms and video uploaders on disseminating reliable information to health seekers.

## Methods

### Data retrieval and collection

**Figure 1:**
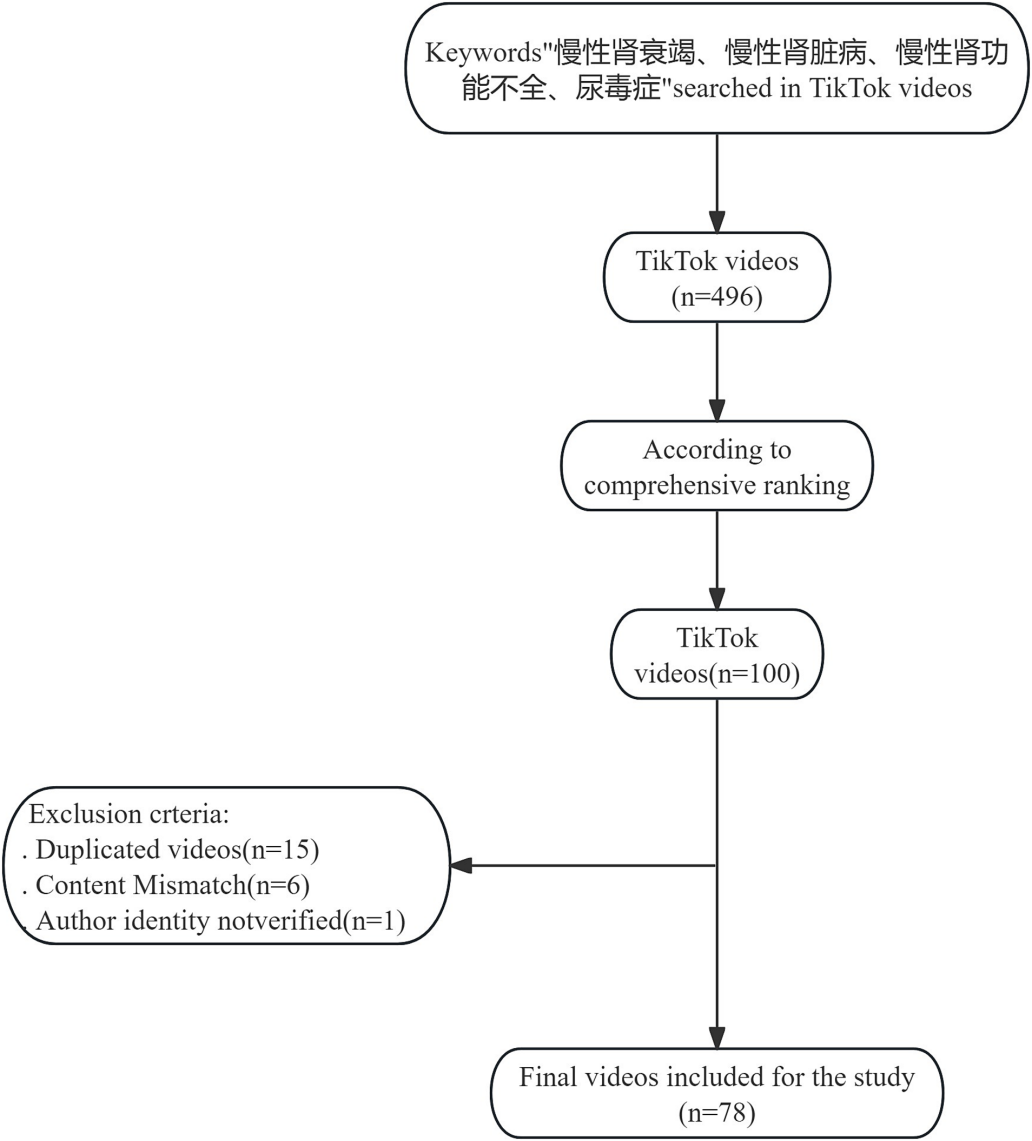
Video retrieval strategy.

To minimize the impact of big data recommendations and personal preferences, a new account was created in the Chinese version of TikTok. We searched in the Chinese version of TikTok with the keywords “慢性肾衰竭” (chronic renal failure), “慢性肾脏病” (chronic kidney disease), “慢性肾功能不全” (chronic renal insufficiency), and “尿毒症” (uremia). According to TikTok’s default settings, videos were sorted by comprehensive ranking, with no restrictions on release time. Comprehensive ranking considers factors such as video completion rate (proportion of viewers who watched more than 5 s), like rate, comment rate, follow rate, and upload time. This ranking helps identify recently uploaded and popular videos (24). The top 100 videos were selected after excluding graphics, untitled videos, non-Chinese videos, and those deleted by the time of scoring. Previous research has shown that videos outside the top 100 do not significantly impact studies (25–27). Content discrepancies, duplicate videos, and those claiming to be medical professionals without verification were removed from the initial set of 100 videos; 78 videos were included in the final study (Figure 1). This cross-sectional study focused on analyzing the content of these selected videos. Characteristics of each video were recorded and analyzed: number of likes, number of comments, number of shares, number of collections, number of downloads, release date, video duration, source of video (publisher), certification status of healthcare professional, and number of followers of the video publisher.

### Video categories

Videos were categorized by source as follows: healthcare professionals (including nephrologists, TCM kidney specialists, and non-nephrology physicians) and non-healthcare professionals (including news organizations, nonprofit organizations, and individual science communicators). Videos were categorized by content as follows: treatment modalities, disease knowledge, lifestyle, and consultation records.

### Assessment methodology

Previous studies have indicated that the JAMA benchmarking standard (28) was not effective in accurately assessing video quality (25, 29). This study used the DISCERN tool (30) and the GQS to evaluate the reliability and quality of video content. The DISCERN tool includes 16 questions divided into three sections: section 1 (questions 1–8) assesses the reliability of the video, section 2 (questions 9–15) evaluates the quality of treatment information, and section 3 (question 16) rates the overall quality. Each question on the DISCERN tool is rated on a 5-point scale, ranging from 1 (very poor) to 5 (excellent). The total score for all 16 questions ranges from 16 to 80, with ratings categorized as 16–26 (very poor), 27–38 (poor), 39–50 (fair), 51–62 (good), and 63–80 (excellent) (31, 32). The GQS evaluates video content on five criteria, with scores ranging from 1 (poor) to 5 (excellent) (Table 1) (33). Although DISCERN was not initially designed for assessing health-related videos, both DISCERN and GQS have been widely adopted for this purpose in recent years (17, 21, 22, 26, 27, 29, 34).

**Table 1:** Description of the Global Quality Score (GQS) 5-point scale used to evaluate videos.

GQS	Description
1	Poor quality; poor flow of the site; most information missing; not at all useful for patients
2	Generally poor quality and poor flow; some information is listed, but many important topics are missing; of very limited use to patients
3	Moderate quality; suboptimal flow; some important information is adequately discussed, but others are poorly discussed; somewhat useful for patients
4	Good quality and generally good flow; most of the relevant information is listed, but some topics are not covered; useful for patients
5	Excellent quality and excellent flow; very useful for the patient

### Evaluation process

Each video was independently evaluated by two raters. Before scoring, the raters reviewed and discussed the official DISCERN tool and GQS scoring guidelines to establish standardized criteria. The consistency between the raters was evaluated using the Kappa test in IBM SPSS Statistics 27. Kappa values less than 0.4 indicate poor agreement, values between 0.4 and 0.6 suggest fair agreement, values between 0.6 and 0.8 reflect high agreement, and values greater than 0.8 denote very high agreement. The interrater reliability for both DISCERN and GQS scores exceeded 0.8, showing good reliability. Spearman and Pearson correlations in IBM SPSS Statistics 27 were used to examine relationships between video features and their DISCERN and GQS scores. The data were statistically analyzed and presented visually using GraphPad Prism version 9.5.

## Results

### Chronic renal failure video features

The 78 videos related to chronic renal failure in this study received a total of 354,293 likes, 88,186 comments, 41,332 collections, 210,303 shares, and 29,436 downloads. The average video duration was 83.18 s, and the average number of days after uploading was 1,269.94 days by the data collection date.

In the sources of videos, 74 (94.87%) were posted by medical professionals, TCM kidney specialists 46 (58.97%), nephrologists 20 (25.64%), and non-nephrology physicians 8 (10.26%). The remaining four videos came from nonmedical professionals, news organizations 2 (2.57%), nonprofit organizations 1 (1.28%), and nonprofessional science communicators 1 (1.28%). Due to the small number of videos sourced by nonmedical professionals, subsequent descriptive statistics and analyses focused on videos sourced by medical professionals. According to video content, disease knowledge is the most dominant video content, which accounts for 40 (51.3%) of all the videos. In addition, the percentage of the remaining content was 19 (24.4%) for treatment modalities, 7 (9.0%) for lifestyle, and 12 (15.4%) for consultation records, respectively (Table 2).

**Table 2:** Descriptive statistics for TikTok videos of different sources and content.

Variable	Duration (seconds), median (IQR)	Likes, median (IQR)	Comments, median (IQR)	Collections, median (IQR)	Shares, median (IQR)	Download, median (IQR)	Days since upload (days), median (IQR)	Videos, *n* (%)
Video source								
TCM kidney specialists	58 (48–81.75)	368.5 (151.75–548)	24.50 (7–58.25)	47.5 (29.50–108)	38.5 (20.50–122.25)	4 (0–18.25)	1271.5 (1254.50–1287.25)	46 (59.0%)
Nephrologists	69.5 (59–103)	539.5 (227.50–1,226)	42 (9.25–72.75)	56 (33.50–94.5)	99.5 (18.50–247.75)	12.5 (4–72.50)	1272.5 (1251.75–1299.25)	20 (25.6%)
Non-nephrology physicians	54.5 (27.25–89.75)	921 (80.75–38,953)	71.5 (7.25–3120.50)	89.5 (13.75–6206.25)	95.5 (30.75–31512.50)	23.5 (3–5187.75)	1297.5 (1270.25–1306.50)	8 (10.3%)
Video content								
Treatment modalities	74 (58–102)	292 (125–979)	15 (5–42)	38 (28–124)	59 (23–120)	4 (0–38)	1,263 (1244–1,286)	19 (24.4%)
Disease knowledge	60 (48.50–81.75)	354.5 (163.25–584.25)	16.5 (7.25–55)	44.5 (24.25–78.75)	45.5 (21–124.50)	5 (1–27.50)	1,273 (1256.25–1293.75)	40 (51.3%)
Lifestyle	75 (65–214)	879 (297–28,941)	42 (10–2,148)	133 (41–7,039)	367 (19–17,242)	27 (6–2,160)	1,282 (1274–1,292)	7 (9.0%)
Consultation records	55 (46.25–60.5)	771.5 (200.75–2227.75)	58.5 (22–664.75)	64 (23.5–391.75)	87 (15.25–364.75)	2.5 (0–250.75)	1282.5 (1254.5–1,307)	12 (15.4%)

### Video quality and reliability

**Figure 2:**
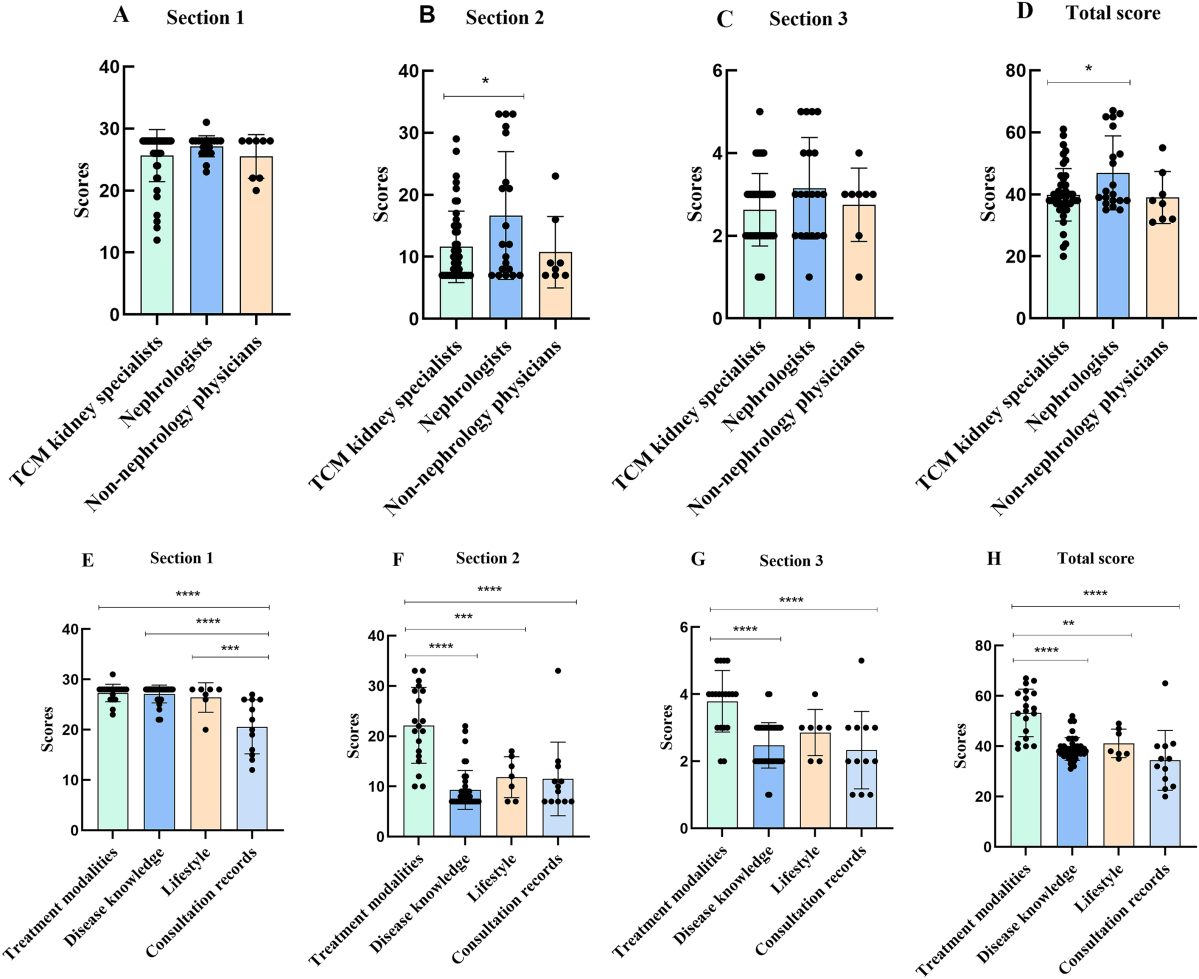
DISCERN scores for TikTok videos of different sources **(A, B, C, D)** and contents **(E, F, G, H)**. *p < 0.05, **p < 0.01, ***p < 0.001, ****p < 0.0001.

We assessed video quality across different sources and content categories using DISCERN and GQS scores. Three sections and the total score of DISCERN were analyzed. In section 1, no significant differences in DISCERN scores were observed among videos from nephrologists, TCM kidney specialists, and non-nephrology physicians. However, DISCERN scores were significantly higher for videos on treatment modalities, disease knowledge, and lifestyle compared to consultation records (*p* < 0.0001, *p* < 0.0001, and *p* = 0.0001). Consultation records were less reliable due to unclear objectives and a lack of supporting sources (questions 1, 2, 4) and because they were single-patient focused and not balanced (question 6). In section 2, DISCERN scores were significantly higher for nephrologists compared to TCM kidney specialists (*p* = 0.03). DISCERN scores were also significantly higher for treatment modalities than for disease knowledge, lifestyle, and consultation records (*p* < 0.0001, *p* = 0.0004, and *p* < 0.0001). This indicates that videos from nephrologists provide better treatment information than those from TCM kidney specialists. In section 3, there were no significant differences in DISCERN scores among videos from TCM kidney specialists, nephrologists, and non-nephrology physicians. However, DISCERN scores for treatment modalities were significantly higher than those for disease knowledge and consultation records (*p* < 0.0001 and *p* < 0.0001). The total score of DISCERN was significantly higher for nephrologists than for TCM kidney specialists (*p* = 0.0188) and for treatment modalities compared to disease knowledge, lifestyle, and consultation records (*p* < 0.0001, *p* = 0.0025, and *p* < 0.0001). Overall, videos on chronic renal failure from nephrologists had better information quality compared to those from TCM kidney specialists. Videos focusing on treatment modalities also had superior quality compared to those focusing on disease knowledge, lifestyle, and consultation records (Figure 2).

**Figure 3:**
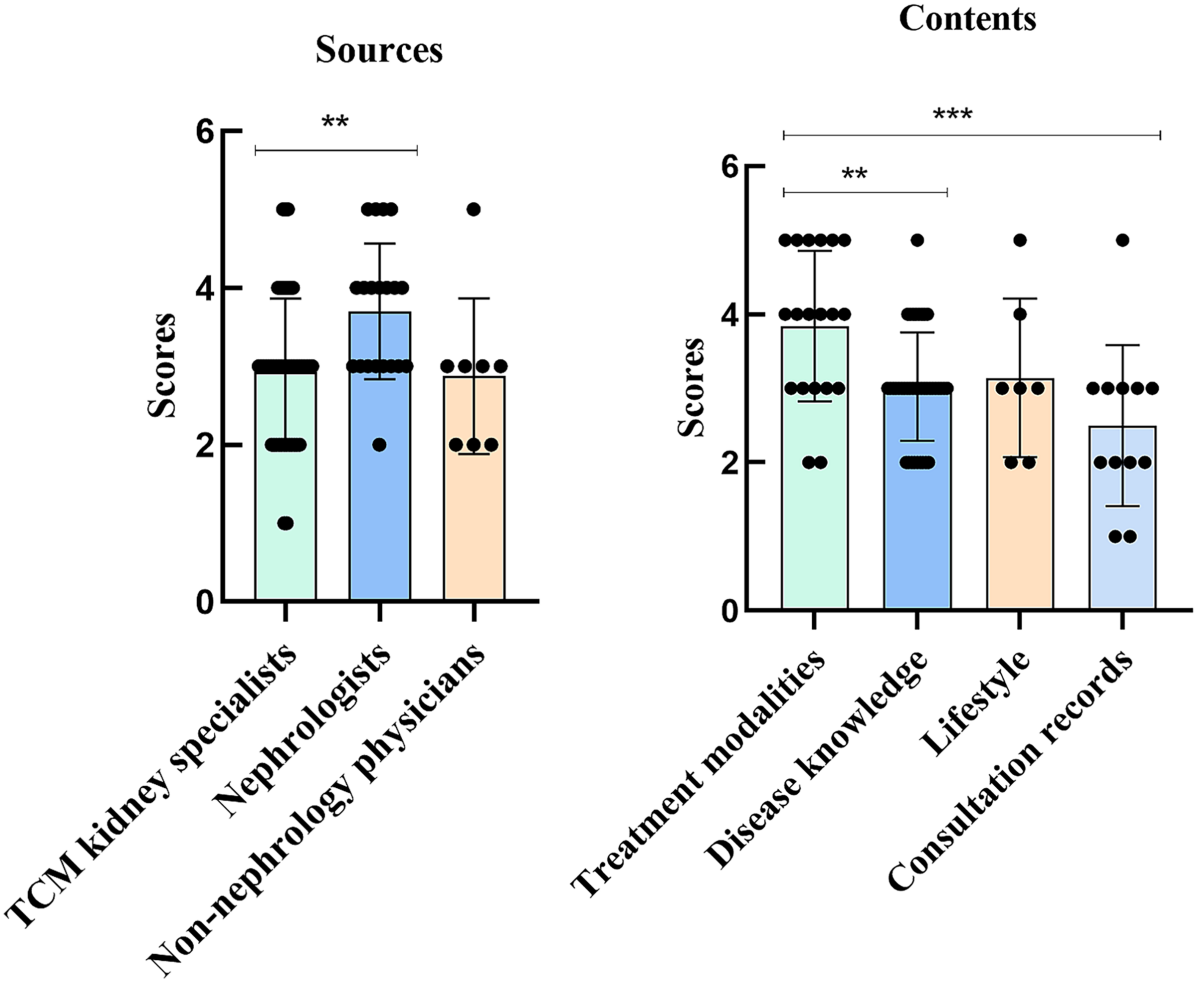
Global Quality Scores (GQS) analysis for TikTok videos of different sources and content. **p < 0.01, ***p < 0.001.

In the GQS analysis, videos from nephrologists scored significantly higher than those from TCM kidney specialists (*p* = 0.0075). Additionally, videos on treatment modalities scored significantly higher than those focused on disease knowledge and consultation records (*p* = 0.0086 and *p* = 0.0007) (Figure 3).

**Figure 4:**
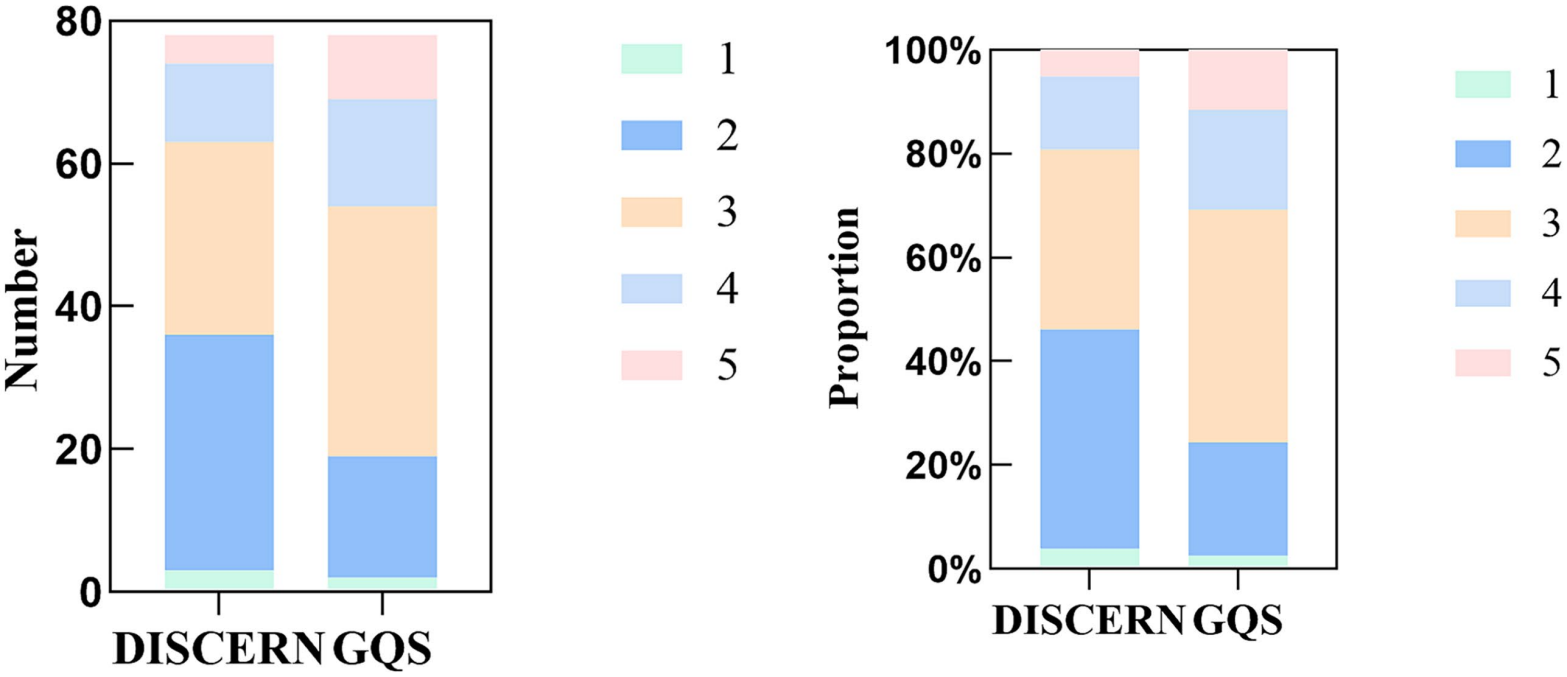
Number and proportion of the 5 levels of DISCERN and the Global Quality Score (GQS).

According to the DISCERN and GQS, chronic renal failure-related videos of quality score on TikTok are not high, mainly at poor (42.31%) and moderate (44.87%). We put these five levels in one-to-one correspondence, and the 5-level scores show some inconsistencies in DISCERN and GQS. This result is different from the previous study (25) (Figure 4; Table 3).

**Table 3:** The 5-level scores of DISCERN and Global Quality Scores (GQS; *n* = 78).

Scores	Value, *n* (%)
DISCERN	
16–26 (very poor)	3 (3.85)
27–38 (poor)	33 (42.31)
39–50 (fair)	27 (34.62)
51–62 (good)	11 (14.10)
63–80 (excellent)	4 (5.12)
GQS	
1 (Poor)	2 (2.57)
2 (Generally poor)	17 (21.79)
3 (Moderate)	35 (44.87)
4 (Good)	15 (19.23)
5 (Excellent)	9 (11.54)

### Correlation analysis

Spearman’s correlation analysis showed that the number of followers was positively correlated with all video characteristics, except that it was not statistically significant with video duration. In addition, the following variables were positively correlated: days since upload with likes (*r* = 0.392, *p* < 0.01), upload with comments (*r* = 0.301, *p* = 0.007), upload with downloads (*r* = 0.307, *p* = 0.006), and upload with shares (*r* = 0.373, *p* = 0.001); likes with comments (*r* = 0.881, *p* < 0.01), likes with downloads (*r* = 0.573, *p* < 0.01), likes with shares (*r* = 0.871, *p* < 0.01), and likes with collections (*r* = 0.732, *p* < 0.01); comments with downloads (*r* = 0.470, *p* < 0.01), comments with shares (*r* = 0.776, *p* < 0.01), and comments with collections (*r* = 0.675, *p* < 0.01); downloads with shares (*r* = 0.608, *p* < 0.01) and downloads with collections (*r* = 0.298, *p* = 0.008); and shares with collections (*r* = 0.725, *p* < 0.01) (Table 4).

**Table 4:** Spearman correlation analysis between video variables.

Variable	Days since upload	Likes	Comments	Download	Shares	Collections	Duration	Number of followers
Days since upload								
*r* value	1							
*p* value	—							
Likes								
*r* value	0.392**	1						
*p* value	0	—						
Comments								
*r* value	0.301**	0.881**	1					
*p* value	0.007	0	—					
Downloads								
*r* value	0.307**	0.573**	0.470**	1				
*p* value	0.006	0	0	—				
Shares								
*r* value	0.373**	0.871**	0.776**	0.608**	1			
*p* value	0.001	0	0	0	—			
Collections								
*r* value	0.142	0.732**	0.675**	0.298**	0.725**	1		
*p* value	0.214	0	0	0.008	0	—		
Duration								
*r* value	−0.195	−0.105	−0.042	−0.047	−0.006	0.168	1	
*p* value	0.087	0.359	0.713	0.681	0.957	0.142	—	
Number of followers								
*r* value	0.308**	0.537**	0.389**	0.300**	0.443**	0.297**	−0.05	1
*p* value	0.006	0	0	0.008	0	0.008	0.667	—

**At the 0.01 level (two-tailed), the correlation is significant.

DISCERN scores were positively correlated with video duration (*r* = 0.384, *p* = 0.001), which aligns with findings from a previous study by Sun Fei on gallstone-related videos on TikTok (25). Similarly, the GQS showed a positive correlation with video duration (*r* = 0.469, *p* < 0.01). However, Jiankun Wang’s research on hypertrophic scarring found no significant correlation between video duration and DISCERN and GQS scores (15). There is no statistically significant relationship between DISCERN and GQS scores and video popularity and number of followers (Table 5).

**Table 5:** Pearson correlation analysis between video quality scores and video variables.

Variable	DISCERN	Global Quality Scores (GQS)
Days since upload		
*r* value	−0.011	−0.14
*p* value	0.927	0.222
Likes		
*r* value	−0.094	0.037
*p* value	0.413	0.75
Comments		
*r* value	−0.111	−0.121
*p* value	0.334	0.293
Downloads		
*r* value	−0.086	0.052
*p* value	0.454	0.651
Shares		
*r* value	−0.104	−0.036
*p* value	0.366	0.752
Collections		
*r* value	0.011	0.179
*p* value	0.925	0.117
Duration		
*r* value	0.384**	0.469**
*p* value	0.001	0
Number of followers		
*r* value	0.065	0.111
*p* value	0.572	0.333

**At the 0.01 level (two-tailed), the correlation is significant.

## Discussion

### Principal findings

This study systematically evaluated the information quality of chronic renal failure videos on TikTok by using the GQS and DISCERN tools and analyzed the characteristics of these videos. Most of the chronic renal failure videos (74/78, 94.87%) were posted by physicians. However, according to DISCERN and GQS, the reliability and quality of videos posted by physicians are poor (35), with mainly poor (33/78, 42.31%) and moderate (35/78, 44.87%), respectively. In addition, it is worth noting that TikTok does not provide mandatory identity verification for video publishers claiming to be doctors. As a result, there may be a possibility of fraudulent identity, and some publishers may exaggerate facts to attract traffic or more views and likes, resulting in the public obtaining false information on social media and leading to misjudgment.

### Factors influencing video popularity

User interactions (likes, shares, and comments) are key indicators of short video quality and reflect video content popularity and user engagement (36, 37). The 78 videos in this study had an average of 4,542 likes, 2,950 shares, 1,131 comments, 530 collections, and 378 downloads. We found a positive correlation between likes and shares, comments, collections, and downloads, suggesting that videos with more likes are also more likely to receive comments, shares, collections, and downloads. Additionally, there was a positive correlation between comments and shares, collections, and downloads, indicating that videos with more comments are more likely to be shared, collected, and downloaded. A positive correlation was also observed between the number of upload days and likes, shares, comments, collections, and downloads, suggesting that older videos are more likely to gain recognition and engagement. However, video duration did not correlate with popularity (25). We found a positive correlation between the number of followers of the video publisher and video popularity, indicating that videos from accounts with more followers are more likely to be liked and recognized. Furthermore, TikTok is more inclined to be an entertainment platform for the public, so the title of the video, the color of the image, and the attractiveness of the first few seconds of the video have an impact on the heat and spread of the video.

### The overall video quality

The median GQS and DISCERN scores for all videos were 3 (IQR 2.75–4) and 39 (IQR 37–46.25), respectively. The reliability and quality of videos related to chronic renal failure on TikTok is generally low, focusing mainly on poor and moderate. However, this stands in stark contrast to previous research on videos related to chronic obstructive pulmonary disease and cosmetic surgery on TikTok (22, 38). Despite the growing number of studies on health-related short videos on TikTok, the quality of these videos remains largely inadequate (39).

In terms of video content, treatment modality-related videos were of higher quality compared to those on disease knowledge, lifestyle, and consultation records. Videos on chronic renal failure from nephrologists were of better quality than those from TCM kidney specialists. In addition, a closer examination of the 16 questions in the DISCERN tool revealed that since the DISCERN tool was originally designed for textual content, therefore the videos all rarely address the source of the information (question 4), when the information was produced (question 5), and the availability of other support and details of the source of the information (question 7).

### Correlation between video quality and video features

We found a positive correlation between the reliability and quality of videos and video duration. The longer the video duration, the higher the video quality and reliability, and medical videos that are too short have more limited and less comprehensive content. The average duration of videos posted by TCM kidney specialists was 69.15 s, and the average duration of videos posted by nephrologists was 101.25 s. The quality of information in videos posted by nephrologists was better than that in videos posted by TCM kidney specialists, which is in line with the results of the study. However, we found no statistically significant relationship between video quality and popularity (likes, shares, and comments), number of followers, which contrasts with previous studies (24, 25, 27, 40). This suggests that more popular videos or those from accounts with more followers are not necessarily of higher quality. In addition, videos with consultation records had the shorter duration (median 55 s, IQR 46.25–60.5), but received the higher median number of likes (771.5, IQR 200.75–2227.75), comments (58.5, IQR 22–664.75), collections (64, IQR 23.5–391.75), and shares (87, IQR 15.25–364.75) compared to videos on disease knowledge and treatment modalities. Previous research has found that video popularity is negatively correlated with video quality, indicating that viewers on TikTok are unable to differentiate between good and bad video quality. Popular videos are more likely to be recommended, resulting in low-quality videos being more likely to be recommended, further exacerbating the disparity between video quality and popularity, and increasing the likelihood that the viewers will be misinformed (25, 27).

### Possible interventions

Previous cross-sectional studies have found that the information quality of videos varies according to the identity of the publisher (38, 41). Our study found that the mean values of likes, comments, and favorites of videos posted by TCM kidney specialists were much higher than those of nephrologists, suggesting that videos posted by TCM kidney specialists were more popular, but the quality of information in videos posted by nephrologists was better than that of TCM kidney specialists. We found that videos featuring non-traditional treatments like traditional Chinese medicine may better align with patients’ expectations and thus prove more popular (42). Therefore, medical video publishers should ensure the professionalism of the content and the quality of the information while avoiding some boring content to improve the attractiveness of medical videos to the public and better disseminate accurate medical information.

Medical video publishers should increase the length of the video when posting medical videos to ensure the completeness and accuracy of the medical video information. The information quality of videos posted by publishers with a high number of followers and popular videos is not necessarily high. However, popular videos are more likely to be recommended, and platforms should recommend medical videos with high information quality. The platform should improve the mechanism of identity verification of medical video publishers, such as checking the graduation certificate or certificate of Licensed Practicing Physician of the publisher, and giving certification marks to video publishers who have passed the professional certification, to facilitate the public’s real access to the health videos published by medical professionals. At the same time, more specialized medical personnel reviewing medical videos can improve the quality of information and reliability.

### Strengths and limitations

This cross-sectional study utilized the DISCERN tool and GQS to evaluate the content, quality, and reliability of chronic renal failure-related videos on TikTok. It also analyzed correlations between video features (likes, comments, video duration, shares, collections, number of followers, downloads, and days since upload) and DISCERN and GQS scores. Video duration was found to be positively correlated with the reliability and quality of videos, and there was no statistically significant relationship between the reliability and quality of videos and popularity and number of followers. While these tools were used for evaluation, other tools, such as the HONcode principles and PEMAT (A/V), could also be valuable. Future research could benefit from combining multiple evaluation tools.

Moreover, the DISCERN tool and GQS were originally designed for evaluating textual content; DISCERN and GQS can only assess the breadth of information contained in a video, not the deeper intrinsic quality of the video content, thus they have limitations when applied to video content. Future research should explore how video titles, image colors, and the initial attractiveness of videos affect their reach and engagement. This study only examined videos from verified uploaders, but some individuals claiming to be doctors were not verified, highlighting the need for future studies to evaluate the quality of information from such unverified sources. Additionally, this study focused solely on Chinese-language videos, so the results may not apply to videos in other languages (e.g., English) related to chronic renal failure on TikTok. The videos and retrieval mechanisms of TikTok may change over time, and analyzing related videos at different times may produce very different results.

## Conclusion

Although chronic renal failure videos on TikTok were mainly posted by doctors and the video content was based on knowledge of the disease, the reliability and quality of the videos were low. The study found a positive correlation between the reliability and quality of videos and video duration, with no statistically significant correlation with popularity or number of followers. Consequently, high-quality videos may receive less attention, while popular videos may be of poorer quality. The speed of speech in most of the videos on TikTok is slower, and the length of the videos is shorter, so the videos involve less information, and the popularized medical content is more limited and not comprehensive enough. Due to the unreliability and low quality of Chinese TikTok videos related to chronic renal failure, such content cannot provide patients with accurate or evidence-based assessments. Therefore, individuals should exercise caution when relying on TikTok for health-related information (24, 41, 43, 44). TikTok is not an appropriate platform for obtaining trustworthy medical knowledge (25). While longer videos may offer patients a broader overview of medical topics, they should not substitute for authoritative sources. For reliable and comprehensive guidance, patients are encouraged to consult professionally produced videos or peer-reviewed documents issued by government health agencies and recognized medical professional bodies.

## Data Availability

The original contributions presented in the study are included in the article/Supplementary material, further inquiries can be directed to the corresponding author.
