# Supplementary material for: Quality and reliability of Chinese short videos on TikTok related to chronic renal failure: cross-sectional study
Source: Front Public Health. 2025 Nov 12;13:1652579. doi: 10.3389/fpubh.2025.1652579 (PMC12646988; doi:10.3389/fpubh.2025.1652579)
Supplement: Supplementary file 1 [file Data_Sheet_1.PDF]

Multimedia Appendix 1

The DISCERN instrument
Section 1: Is the publication reliable?
Question 1: Are the aims clear?
Question 2: Does it achieve its aims?
Question 3: Is it relevant?
Question 4: Is it clear what sources of information were used to compile the publication (other than the author or producer)?
Question 5: Is it clear when the information used or reported in the publication was produced?
Question 6: Is it balanced and unbiased?
Question 7: Does it provide details of additional sources of support and information?
Question 8: Does it refer to areas of uncertainty?
Section 2: How good is the quality of information on treatment choices?
Question 9: Does it describe how each treatment works?
Question 10: Does it describe the benefits of each treatment?
Question 11: Does it describe the risks of each treatment?
Question 12: Does it describe what would happen if no treatment is used?
Question 13: Does it describe how the treatment choices affect overall quality of life?
Question 14: Is it clear that there may be more than one possible treatment choice?
Question 15: Does it provide support for shared decision-making?
Section 3: Overall rating
Question 16: Based on the answers to all of the above questions, rate the overall quality of the publication as a source of information about treatment choices.
