# Supplementary material for: Quality and reliability of Chinese short videos on TikTok related to chronic renal failure: cross-sectional study
Source: Front Public Health. 2025 Nov 12;13:1652579. doi: 10.3389/fpubh.2025.1652579 (PMC12646988; doi:10.3389/fpubh.2025.1652579)
Supplement: Supplementary file 2 [file Data_Sheet_2.PDF]

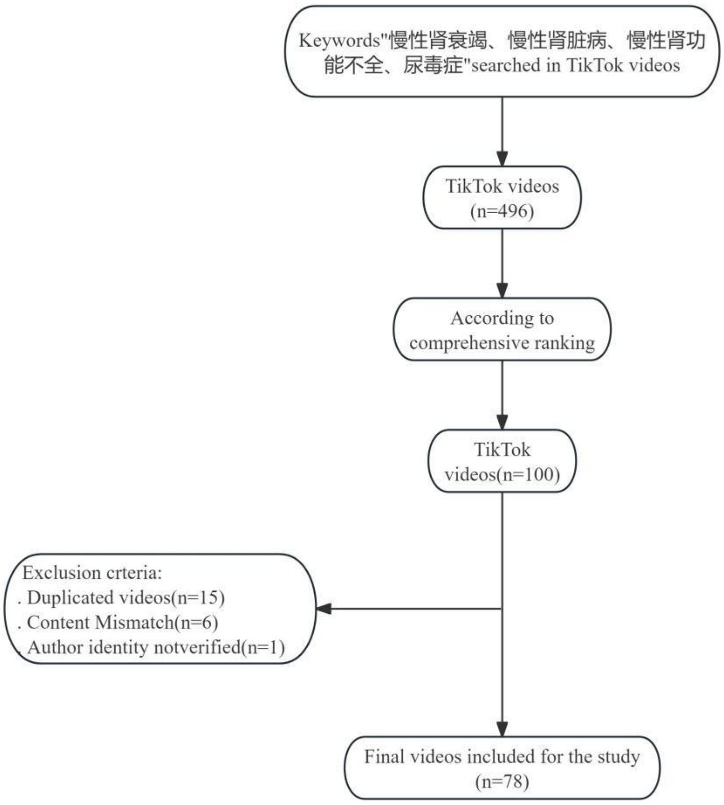

Figure. 1 Video retrieval strategy.

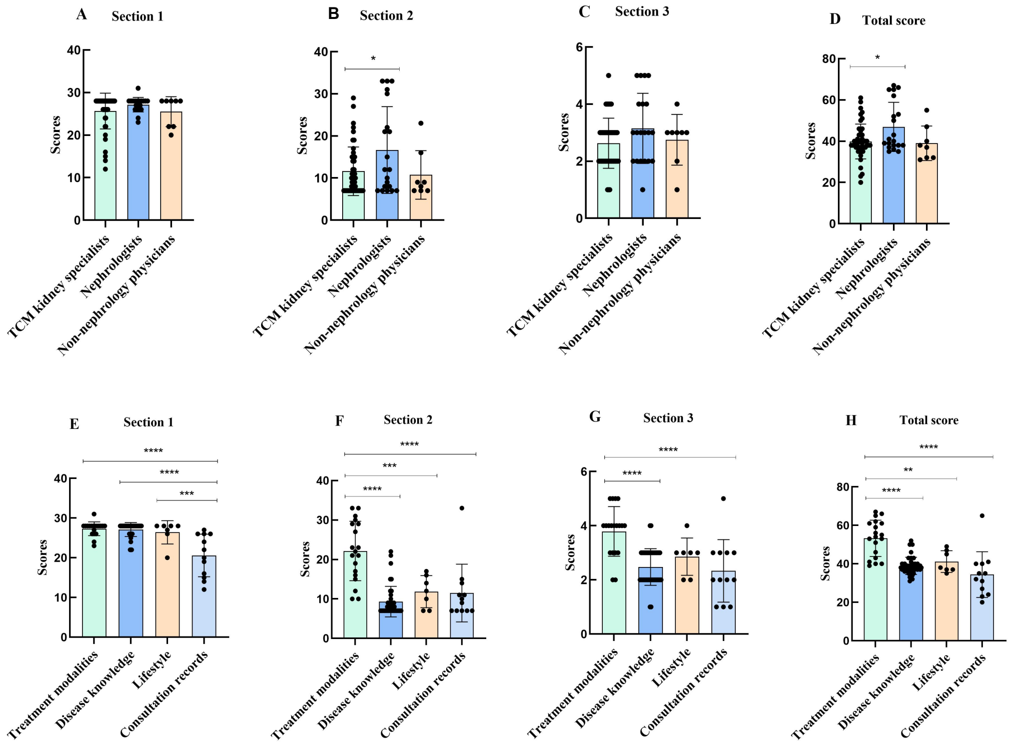

Figure 2. DISCERN scores for TikTok videos of different sources (A, B, C, D) and contents (E, F, G, H). **P*<.05, ***P*<.01, ****P*<.001,*****P*<.0001.

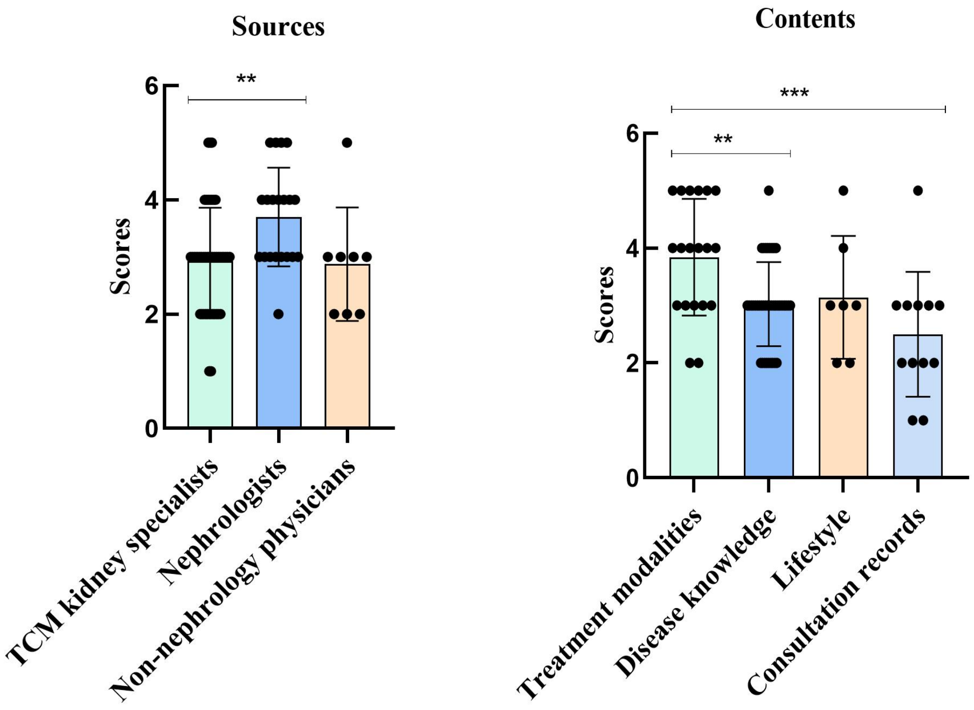

Figure3. Global Quality Scores(GQS) analysis for TikTok videos of different sources and content.

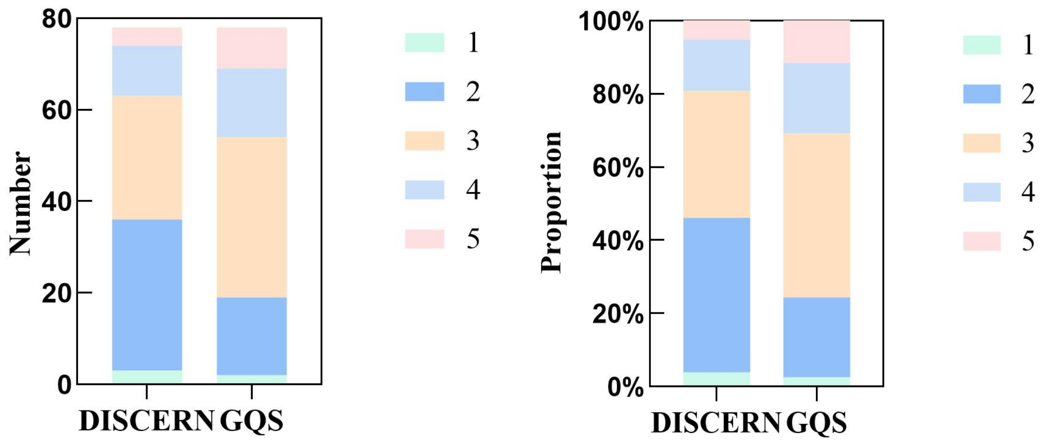

Figure 4.Number and proportion of the 5 levels of DISCERN and the Global Quality Score (GQS).
